# Age‐ and Sex‐Specific Percentile Curves for the FM/FFM Ratio Using Data From the Canadian Longitudinal Study on Aging

**DOI:** 10.1002/jcsm.70399

**Published:** 2026-10-08

**Authors:** Chris Kim, Claire E. Cook, Hailey R. Banack

**Affiliations:** ^1^ Epidemiology Division, Dalla Lana School of Public Health University of Toronto Toronto Ontario Canada

**Keywords:** aging, CLSA, fat mass, fat‐free mass, sarcopenic obesity

## Abstract

**Background:**

Sarcopenic obesity is associated with a significant increase in morbidity and mortality, particularly in older adults. Common measures of sarcopenic obesity consider obesity and sarcopenia independently, despite their potentially synergistic harmful effects. The fat mass to fat‐free mass ratio (FM/FFM) is an integrated measure of sarcopenic obesity that provides more useful information than measures that consider sarcopenia and obesity separately. The objectives of this study are to develop age‐ and sex‐specific FM/FFM percentile curves for older adults and describe age‐ and sex‐related patterns of FM/FFM percentiles.

**Methods:**

Data from the Canadian Longitudinal Study on Aging (CLSA) were used (*n* = 28 791). FM/FFM was calculated as the ratio of total body fat mass (kg) to total body lean mass (kg) from dual‐energy X‐ray absorptiometry (DXA) scan. Weighted age‐ and sex‐specific FM/FFM curves for the 1st, 5th, 25th, 50th, 75th, 95th and 99th percentiles were calculated using the LMS method. LMS provides a summary of the changing distribution of the measurement of interest by the three curves, lambda for the skewness (L), mu for the median (M) and sigma for the coefficient of variation (S). Weighted percentile curves were plotted with age (45–86 years) on the *x*‐axis and FM/FFM on the *y*‐axis for males and females, respectively.

**Results:**

Mean age of the CLSA at baseline was 59.6 ± 10.2 years, and 47.4% were males. For men, FM/FFM percentiles appear to increase slowly with aging and then stabilise after age 70. For women, FM/FFM percentiles appear to start at a higher value and increase more rapidly with aging, stabilising but then showing a stronger downward trend than males after age 65. At the 50th percentile, the maximum FM/FFM value is 0.42 at age 86 years for males and 0.71 at age 68 years for females.

**Conclusions:**

The present study highlights the important age‐ and sex‐related differences in FM/FFM percentiles in older adults. The FM/FFM curves could be used as a tool for identifying older adults with sarcopenic obesity and examining health risks associated with sarcopenic obesity.

## 1 Introduction

Sarcopenic obesity is an emerging public health threat in the context of the obesity epidemic and demographic shifts towards aging populations [1, 2]. Sarcopenic obesity is defined as the coexistence of obesity and sarcopenia. However, there is currently a lack of consensus about how to operationalise and measure sarcopenic obesity in clinical settings and population health research [1, 3, 4]. For example, the prevalence of sarcopenic obesity has been reported to range from 3% to over 20%, depending on the specific definition, diagnostic criteria and cutoffs being used [5]. This is a major barrier for identification and treatment of individuals with sarcopenic obesity [3]. Sarcopenic obesity is associated with an increased risk of adverse health outcomes in older adults compared with either obesity or sarcopenia alone [4, 6, 7]. Prior research has demonstrated that sarcopenic obesity is associated with falls, fractures, cardiometabolic disease, worse cancer outcomes and all‐cause mortality [2, 4].

Sarcopenia is a geriatric syndrome that is often progressive and generalised, characterised by decreasing muscle mass and decline in strength and function with age [8]. Sarcopenia is commonly measured using appendicular skeletal muscle mass adjusted by weight (ASM/W) or height in meters squared (ASM/h^2^) [5]. Clinically, obesity is defined as having excess fat accumulation in the body [9]. The World Health Organization and most clinical societies use body mass index (BMI) greater than or equal to 30 kg/m^2^ to define obesity [9]. Recent consensus statements have recommended the use of direct measurement of fat mass such as whole‐body dual X‐ray absorptiometry (DXA) for diagnosing obesity [10].

Researchers studying sarcopenic obesity typically define sarcopenia and obesity independently (i.e., presence of low ASM and high BMI) [3]. This is despite research demonstrating a potential synergistic effect of obesity and sarcopenia due to pathophysiological interactions [3]. There is limited research examining an integrated measure of sarcopenic obesity that simultaneously considers both fat mass and muscle mass [3]. One validated measure that includes both sarcopenia and obesity is the fat mass to fat‐free mass ratio (FM/FFM) [3, 5]. FM/FFM was first proposed as a measure of sarcopenic obesity by Prado and colleagues in 2012 [11]. FM/FFM describes the impact of both FM and FFM distribution on overall body composition and within‐individual variation by conceptualising two opposing traits of homeostasis [6]. These traits are (1) metabolic load, or the magnitude of an insult on a system, measured via FM, and (2) metabolic capacity, or the ability of the system to offset the insult, measured via FFM [6].

There is growing evidence supporting the associations between FM/FFM and health outcomes in older adults [12, 13, 14, 15]. However, little is known about the distribution of FM/FFM at the population level. Understanding the distribution of FM/FFM in older adults could inform public health strategies for targeted interventions and healthy aging. Examining the descriptive epidemiology of sarcopenic obesity is an important step forward to understand the distribution of sarcopenic obesity at a population level and among demographic subgroups [16, 17]. In the present study, we developed age‐ and sex‐specific FM/FFM percentile curves to measure and describe the distribution of sarcopenic obesity in older adults. To our knowledge, this work is the first‐ever description of sarcopenic obesity at a population level in older adults in Canada using FM/FFM.

## 2 Methods

### 2.1 Study Population

The present study used baseline data from the Canadian Longitudinal Study on Aging (CLSA). The CLSA is a population‐based longitudinal study of 51 338 adults aged 45–85 years from all 10 Canadian provinces. The goal of the study is to understand and address the needs of an aging population [18]. Of the 51 338 adults, 30 097 adults were selected to be part of the comprehensive cohort, including in‐person measurements. The comprehensive cohort participants were randomly selected from those who lived within 25–50 km of the 11 data collection sites and participated in in‐depth physical assessments, interviews and provided blood and urine samples [18]. Baseline data collection began in 2011 and was completed in 2015.

Our analytic cohort comprised a subsample from the CLSA Comprehensive cohort who completed whole body DXA examination at baseline. DXA was performed using Hologic Discovery A (Marlborough, MA, United States) [19]. Exclusion criteria for the DXA examination are height greater than 188 cm and/or weight greater than 204 kg, pregnancy and participation in a nuclear medicine study in the last 7 days [19]. DXA scan measured total fat mass (kg), total lean mass (kg), fat and lean tissue in the extremities and trunk and total and regional bone mineral density.

### 2.2 Calculation of FM/FFM

FM/FFM was calculated based on the approach developed by Prado and colleagues [11]: total fat mass (kg)/total lean mass (kg). Consistent with prior research, total lean mass was used as a surrogate for FFM on the basis that lean mass is equivalent to a composite of FFM and lipid in cellular membranes [20]. This is an appropriate surrogate given that cellular lipid content only accounts for a very small percentage of total body weight (maximum 3% in men and 5% in women) [20].

### 2.3 Statistical Analysis

Descriptive statistics were calculated by age, sex, race, marital status, education and household income. Consistent with guidance from the CLSA, inflation weights were used in the descriptive analysis [18]. Inflation weights, whose sum approximates the size of the target Canadian population, were used for descriptive analyses to obtain population‐representative estimates from the study sample. Using the inflation weights, continuous variables were presented as weighted means with standard deviations and categorical variables were presented as weighted proportions and their 95% confidence intervals.

Age‐ and sex‐specific FM/FFM percentile curves were developed using the LMS method developed by Cole and Green [21]. A percentile refers to the percentage of observations that fall at a given value, comparing the score of an individual with others in the population [22]. Percentile scores can be used to determine the relative position of an individual within the population, facilitating comparison with others in the same population. For example, an individual in the 75th percentile has an FM/FFM value that is greater than 75% of individuals in the population whereas an individual in the 10th percentile has an FM/FFM value that is greater than only 10% of individuals in the population. Percentiles are commonly known as a tool in paediatrics as they are used to generate growth charts and are easily interpretable in clinical settings [22]. CLSA analytic weights were used to generate the FM/FFM percentile curves. The analytic weights are rescaled versions of the inflation weights that preserve the relative distribution of participants; they maintain the representativeness of the sample while avoiding artificial inflation of precision that may occur when inflation weights are used in regression‐based modelling approaches, including the LMS method [18, 21]. Weighted percentile curves were used to describe the distribution of sarcopenic obesity in the Canadian population.

The LMS method provides a summary of the changing distribution of a measurement (FM/FFM in this case) by three curves: skewness expressed as a power in the Box–Cox transformation and represented by the letter lambda (λ), the median represented by the letter mu (μ) and the coefficient of variation represented by the letter sigma (ς), hence its name, LMS [21]. In doing so, this method allows for percentiles to be estimated in a continuous manner as well as their corresponding *z*‐scores [23]. A *z*‐score, defined as the deviation of an individual value from the mean of the reference population divided by the standard deviation of the same reference population, has a direct relationship with percentiles and allows for calculation of exact percentiles, which may be preferred by clinicians for greater precision [23].

At each age grouping of interest, a system of 12 equations, each representing one of the percentiles of interest (i.e., 1st‐99th percentiles), was generated by specifying the LMS transformation equation at these percentiles:

$$X=\mu \;{\left(1+\mathrm{\lambda \varsigma Z}\right)}^{\frac{1}{\lambda }},\lambda \neq 0$$

or

$$X=\mu \exp\left(\mathrm{\varsigma Z}\right),\lambda =0$$

These equations were solved for the three LMS parameters by minimising the sum of squared errors, where X denotes FM/FFM and Z denotes the *z*‐score corresponding to the percentile of the value of X. Degrees of freedom for the LMS parameters were selected by fitting models with 3° to 6° of freedom and comparing the Bayesian information criterion (BIC) with the goal of balancing curve smoothness and model fit. These candidate degrees of freedom were chosen based on guidance from Cole and Green [21]. Using the above equation and the estimated LMS parameters, *z*‐scores corresponding to the percentile estimates for 1st, 5th, 25th, 50th, 75th, 95th and 99th percentiles were derived for each age grouping, stratified into males and females. A final set of percentile curves were generated based on FM/FFM estimates at these percentiles for each sex. To assess the model fit and normality of the generated percentile curves, observed versus predicted percentile plots and Q‐Q plot of *z* scores were used. As a sensitivity analysis to assess the potential impact of applying sampling weights, unweighted FM/FFM percentile curves were also created.

All analyses were performed using R Studio 2022.07.1.

## 3 Results

The analytic cohort was comprised of 28 791 participants. We excluded 1306 CLSA participants without a DXA scan at baseline. The weighted mean age of the study sample was 59.6 ± 10.2 years (Table 1). In this study sample, 47.7% [46.6%, 48.8%] were men. Most participants identified as being white (92.1% [91.4%, 92.8%]), were married or in a common‐law relationship (74.8% [73.8%, 75.7%]) and had income between $50 000 and $100 000 (31.4% [30.3%, 32.4%]). Many participants had a bachelor's degree (13.5% [13.0%, 14.0%]) or higher (11.6% [11.1%, 12.1%]).

**TABLE 1:** Demographics of the study population estimated using CLSA inflation weights.

Variable	Weighted point estimate	95% CI
Age (mean ± SD)	59.6 ± 10.2	[59.3, 59.8]
Sex (%)		
Male	47.7%	[46.6%, 48.8%]
Female	52.3%	[51.2%, 53.4%]
Race (%)		
White	92.1%	[91.4%, 92.8%]
Black	1.3%	[1.0%, 1.6%]
Asian (West Asian and Middle Eastern, South Asian, East and Southeast Asian)	3.6%	[3.1%, 4.1%]
Hispanic/American	0.5%	[0.3%, 0.8%]
Other	0.6%	[0.5%, 0.8%]
Multiple	1.7%	[1.4%, 1.9%]
Do not know/refused	0.2%	[0.1%, 0.3%]
Marital/partner status (%)		
Single, never married or never lived with a partner	8.5%	[7.9%, 9.1%]
Married/living with a partner in a common‐law relationship	74.8%	[73.8%, 75.7%]
Widowed	6.2%	[5.7%, 6.7%]
Divorced	8.0%	[7.5%, 8.6%]
Separated	2.5%	[2.1%, 2.9%]
Refused	0%	[0%, 0.1%]
Education highest degree (%)		
No postsecondary degree, certificate or diploma	8.9%	[8.4%, 9.5%]
Trade certificate or diploma from a vocational school or apprenticeship training	13.5%	[12.7%, 14.3%]
Nonuniversity certificate or diploma from a community college, CEGEP, school of nursing, etc.	19.1%	[18.3%, 19.9%]
University certificate below bachelor's level	4.5%	[4.1%, 4.9%]
Bachelor's degree	13.5%	[13.0%, 14.0%]
University degree or certificate above bachelor's degree	11.6%	[11.1%, 12.1%]
Other	0.2%	[0.1%, 0.3%]
Do not know/no answer	0%	[0%, 0%]
Missing	28.6%	[27.3%, 29.9%]
Total household income (%)		
Less than $20 000	6.0%	[5.4%, 6.7%]
$20 000 or more, but less than $50 000	20.9%	[19.9%, 21.9%]
$50 000 or more, but less than $100 000	31.4%	[30.3%, 32.4%]
$100,00 or more, but less than $150 000	19.2%	[18.3%, 20.1%]
$150 000 or more	16.6%	[15.8%, 17.3%]
Do not know/no answer	2.3%	[1.9%, 2.6%]
Refused	3.7%	[3.3%, 4.1%]

*Note:* Unweighted sample size *n* = 28 791; sample size using CLSA inflation weights = 3 603 512.

Abbreviations: CI, confidence interval.

Age‐ and sex‐specific FM/FFM percentile curves are presented for males and females aged 45–86 years in Figure 1. FM/FFM values are consistently lower for males than females, and, in men, percentile curves appear to change less across the lifespan compared with women. In men, FM/FFM values do not exceed 0.75, whereas in women, they are greater than 1.1 between age 55 and 65 years. For females, FM/FFM percentiles start at a higher value and increase more rapidly with aging. The percentile curves in women also demonstrate a sharper downward trend than males in older age groups. For males, the maximum FM/FFM value of the 95th percentile is 0.62 at age 70 years, whereas for females, it is 0.99 at age 65 years (Tables 2 and 3). At the 50th percentile, the maximum FM/FFM value is 0.42 at age 86 years for males and 0.71 at age 68 years for females. At the 25th percentile, the maximum value is 0.36 at age 86 years for males and 0.60 at age 70 years for females. FM/FFM *z*‐score values also change with age for both sexes (Tables 4 and 5).

**FIGURE 1:**
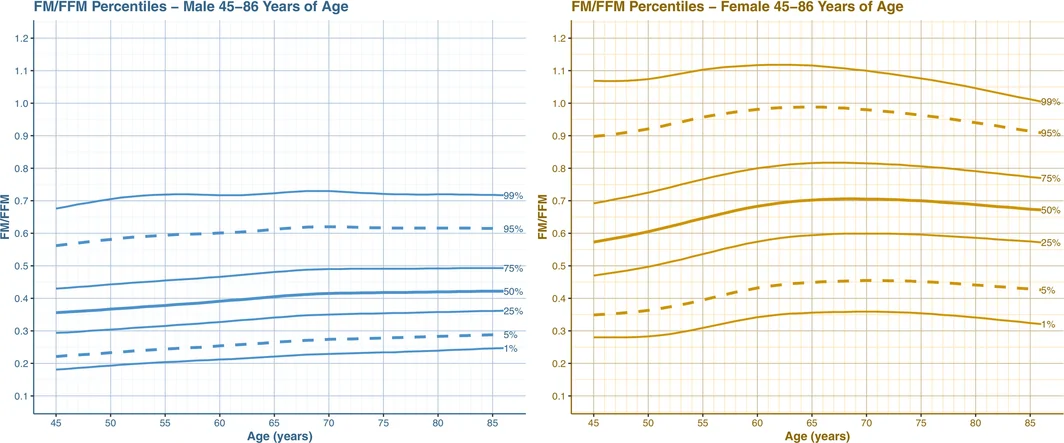
FM/FFM percentile curves.

**TABLE 2:** Age‐specific LMS parameters and FM/FFM percentiles for males.

Age	L	M	S	1st	5th	25th	50th	75th	95th	99th
45	0.076	0.356	0.283	0.181	0.221	0.294	0.356	0.43	0.562	0.676
46	0.05	0.358	0.282	0.183	0.224	0.295	0.358	0.432	0.566	0.682
47	0.026	0.36	0.281	0.186	0.226	0.297	0.36	0.435	0.57	0.689
48	0.003	0.362	0.28	0.188	0.228	0.3	0.362	0.437	0.574	0.694
49	−0.017	0.364	0.279	0.191	0.23	0.302	0.364	0.44	0.578	0.7
50	−0.034	0.367	0.278	0.193	0.233	0.304	0.367	0.443	0.581	0.705
51	−0.048	0.369	0.277	0.196	0.235	0.306	0.369	0.445	0.585	0.71
52	−0.058	0.371	0.275	0.198	0.238	0.309	0.371	0.448	0.588	0.713
53	−0.063	0.374	0.274	0.2	0.24	0.311	0.374	0.45	0.59	0.716
54	−0.064	0.376	0.272	0.202	0.242	0.313	0.376	0.452	0.592	0.718
55	−0.061	0.378	0.271	0.204	0.244	0.315	0.378	0.455	0.594	0.719
56	−0.054	0.381	0.269	0.205	0.246	0.318	0.381	0.457	0.596	0.72
57	−0.042	0.383	0.268	0.207	0.248	0.32	0.383	0.459	0.597	0.72
58	−0.027	0.385	0.266	0.209	0.249	0.322	0.385	0.461	0.598	0.719
59	−0.008	0.388	0.264	0.21	0.251	0.325	0.388	0.464	0.599	0.718
60	0.013	0.391	0.262	0.212	0.254	0.327	0.391	0.466	0.601	0.717
61	0.032	0.394	0.26	0.213	0.256	0.33	0.394	0.469	0.602	0.717
62	0.05	0.396	0.259	0.215	0.258	0.333	0.396	0.472	0.604	0.717
63	0.063	0.399	0.257	0.217	0.26	0.335	0.399	0.475	0.606	0.719
64	0.072	0.402	0.256	0.219	0.262	0.338	0.402	0.478	0.609	0.721
65	0.077	0.405	0.255	0.221	0.265	0.341	0.405	0.481	0.612	0.723
66	0.078	0.408	0.254	0.223	0.267	0.343	0.408	0.483	0.615	0.726
67	0.078	0.41	0.252	0.225	0.269	0.346	0.41	0.486	0.617	0.728
68	0.079	0.412	0.251	0.227	0.271	0.348	0.412	0.488	0.619	0.73
69	0.081	0.414	0.25	0.228	0.272	0.349	0.414	0.489	0.62	0.73
70	0.085	0.415	0.249	0.229	0.274	0.35	0.415	0.49	0.62	0.73
71	0.091	0.416	0.247	0.23	0.275	0.351	0.416	0.49	0.62	0.728
72	0.098	0.416	0.246	0.231	0.275	0.352	0.416	0.491	0.619	0.726
73	0.104	0.417	0.245	0.232	0.276	0.353	0.417	0.491	0.618	0.724
74	0.108	0.417	0.243	0.233	0.277	0.353	0.417	0.491	0.617	0.723
75	0.11	0.418	0.242	0.234	0.278	0.354	0.418	0.491	0.617	0.721
76	0.108	0.418	0.241	0.234	0.279	0.355	0.418	0.491	0.616	0.72
77	0.104	0.418	0.24	0.236	0.28	0.355	0.418	0.491	0.616	0.72
78	0.098	0.419	0.239	0.237	0.281	0.356	0.419	0.491	0.616	0.719
79	0.089	0.419	0.238	0.238	0.282	0.357	0.419	0.492	0.616	0.719
80	0.079	0.42	0.236	0.239	0.283	0.358	0.42	0.492	0.616	0.72
81	0.069	0.42	0.235	0.241	0.284	0.358	0.42	0.492	0.616	0.72
82	0.058	0.421	0.234	0.242	0.285	0.359	0.421	0.493	0.616	0.719
83	0.049	0.421	0.233	0.243	0.286	0.36	0.421	0.493	0.616	0.719
84	0.039	0.422	0.231	0.245	0.287	0.361	0.422	0.493	0.615	0.718
85	0.031	0.422	0.23	0.246	0.288	0.361	0.422	0.493	0.615	0.718
86	0.022	0.422	0.229	0.247	0.289	0.362	0.422	0.493	0.614	0.717

**TABLE 3:** Age‐specific LMS parameters and FM/FFM percentiles for females.

Age	L	M	S	1st	5th	25th	50th	75th	95th	99th
45	0.209	0.573	0.286	0.28	0.349	0.47	0.573	0.692	0.898	1.069
46	0.256	0.579	0.285	0.28	0.351	0.475	0.579	0.698	0.901	1.068
47	0.303	0.585	0.284	0.28	0.353	0.48	0.585	0.705	0.905	1.068
48	0.347	0.591	0.283	0.28	0.356	0.485	0.591	0.711	0.91	1.069
49	0.388	0.598	0.281	0.281	0.359	0.491	0.598	0.718	0.915	1.071
50	0.422	0.605	0.279	0.283	0.363	0.497	0.605	0.725	0.921	1.074
51	0.451	0.613	0.277	0.286	0.368	0.504	0.613	0.733	0.927	1.079
52	0.472	0.621	0.274	0.29	0.374	0.512	0.621	0.741	0.935	1.085
53	0.488	0.629	0.272	0.296	0.38	0.519	0.629	0.75	0.942	1.091
54	0.5	0.638	0.268	0.302	0.387	0.528	0.638	0.758	0.95	1.097
55	0.511	0.646	0.264	0.309	0.395	0.536	0.646	0.766	0.957	1.103
56	0.522	0.654	0.26	0.316	0.403	0.544	0.654	0.774	0.963	1.107
57	0.535	0.662	0.256	0.323	0.411	0.553	0.662	0.781	0.968	1.111
58	0.549	0.67	0.252	0.33	0.419	0.56	0.67	0.788	0.973	1.113
59	0.565	0.677	0.248	0.336	0.425	0.568	0.677	0.794	0.977	1.115
60	0.582	0.683	0.245	0.342	0.432	0.574	0.683	0.8	0.981	1.117
61	0.601	0.688	0.242	0.346	0.437	0.58	0.688	0.804	0.984	1.118
62	0.62	0.693	0.239	0.35	0.441	0.585	0.693	0.808	0.986	1.118
63	0.641	0.697	0.237	0.353	0.445	0.589	0.697	0.812	0.987	1.118
64	0.661	0.7	0.236	0.354	0.447	0.592	0.7	0.814	0.988	1.117
65	0.681	0.702	0.234	0.356	0.449	0.594	0.702	0.816	0.988	1.116
66	0.7	0.704	0.232	0.357	0.451	0.596	0.704	0.817	0.988	1.113
67	0.717	0.705	0.231	0.358	0.452	0.598	0.705	0.817	0.986	1.11
68	0.733	0.706	0.23	0.358	0.453	0.599	0.706	0.817	0.985	1.107
69	0.748	0.706	0.228	0.359	0.454	0.599	0.706	0.816	0.982	1.103
70	0.762	0.705	0.227	0.359	0.455	0.599	0.705	0.815	0.98	1.1
71	0.776	0.705	0.226	0.359	0.455	0.599	0.705	0.814	0.977	1.095
72	0.79	0.704	0.225	0.358	0.454	0.599	0.704	0.812	0.974	1.091
73	0.806	0.703	0.224	0.357	0.454	0.598	0.703	0.811	0.971	1.086
74	0.822	0.701	0.224	0.356	0.453	0.597	0.701	0.808	0.967	1.081
75	0.839	0.7	0.223	0.354	0.451	0.596	0.7	0.806	0.963	1.076
76	0.858	0.698	0.223	0.352	0.45	0.594	0.698	0.803	0.959	1.071
77	0.877	0.695	0.222	0.349	0.448	0.592	0.695	0.801	0.955	1.065
78	0.898	0.693	0.222	0.347	0.446	0.59	0.693	0.798	0.95	1.059
79	0.919	0.691	0.221	0.344	0.443	0.588	0.691	0.794	0.945	1.053
80	0.942	0.688	0.221	0.341	0.441	0.586	0.688	0.791	0.94	1.046
81	0.967	0.685	0.22	0.338	0.439	0.584	0.685	0.787	0.935	1.039
82	0.992	0.682	0.22	0.334	0.436	0.581	0.682	0.784	0.93	1.032
83	1.018	0.68	0.219	0.331	0.434	0.579	0.68	0.78	0.924	1.025
84	1.045	0.677	0.219	0.328	0.431	0.577	0.677	0.777	0.919	1.018
85	1.071	0.674	0.218	0.324	0.429	0.575	0.674	0.773	0.914	1.012
86	1.098	0.672	0.218	0.321	0.426	0.572	0.672	0.77	0.909	1.005

**TABLE 4:** Age‐specific LMS parameters and FM/FFM *z* scores for males.

Age	L	M	S	−3.0 SD	−2.5 SD	−2.0 SD	−1.5 SD	−1.0 SD	−0.5 SD	0.5 SD	1.0 SD	1.5 SD	2.0 SD	2.5 SD	3.0 SD
45	0.076	0.356	0.283	0.148	0.172	0.199	0.231	0.267	0.309	0.41	0.471	0.54	0.619	0.709	0.81
46	0.05	0.358	0.282	0.151	0.174	0.202	0.233	0.269	0.31	0.412	0.473	0.544	0.624	0.716	0.82
47	0.026	0.36	0.281	0.153	0.177	0.204	0.235	0.271	0.313	0.414	0.476	0.547	0.629	0.722	0.829
48	0.003	0.362	0.28	0.156	0.179	0.206	0.238	0.273	0.315	0.416	0.479	0.551	0.634	0.729	0.838
49	−0.017	0.364	0.279	0.159	0.182	0.209	0.24	0.276	0.317	0.419	0.482	0.555	0.638	0.735	0.847
50	−0.034	0.367	0.278	0.161	0.184	0.211	0.242	0.278	0.319	0.421	0.485	0.558	0.643	0.741	0.855
51	−0.048	0.369	0.277	0.164	0.187	0.214	0.245	0.28	0.322	0.424	0.488	0.561	0.647	0.746	0.861
52	−0.058	0.371	0.275	0.166	0.189	0.216	0.247	0.283	0.324	0.426	0.49	0.564	0.65	0.749	0.865
53	−0.063	0.374	0.274	0.168	0.191	0.218	0.249	0.285	0.326	0.429	0.493	0.567	0.653	0.752	0.869
54	−0.064	0.376	0.272	0.17	0.193	0.22	0.251	0.287	0.328	0.431	0.495	0.569	0.655	0.754	0.87
55	−0.061	0.378	0.271	0.171	0.195	0.222	0.253	0.289	0.331	0.433	0.497	0.571	0.656	0.755	0.871
56	−0.054	0.381	0.269	0.173	0.196	0.224	0.255	0.291	0.333	0.436	0.499	0.573	0.657	0.756	0.869
57	−0.042	0.383	0.268	0.174	0.198	0.226	0.257	0.293	0.335	0.438	0.501	0.574	0.658	0.755	0.867
58	−0.027	0.385	0.266	0.175	0.199	0.227	0.259	0.296	0.337	0.44	0.503	0.575	0.658	0.754	0.863
59	−0.008	0.388	0.264	0.176	0.201	0.229	0.261	0.298	0.34	0.443	0.505	0.577	0.658	0.752	0.859
60	0.013	0.391	0.262	0.177	0.202	0.231	0.263	0.3	0.343	0.445	0.508	0.578	0.659	0.75	0.854
61	0.032	0.394	0.26	0.178	0.204	0.233	0.266	0.303	0.345	0.448	0.51	0.58	0.659	0.749	0.851
62	0.05	0.396	0.259	0.18	0.205	0.235	0.268	0.306	0.348	0.451	0.513	0.582	0.661	0.749	0.849
63	0.063	0.399	0.257	0.181	0.207	0.237	0.27	0.308	0.351	0.454	0.515	0.585	0.663	0.75	0.848
64	0.072	0.402	0.256	0.183	0.209	0.239	0.273	0.311	0.354	0.457	0.518	0.588	0.665	0.752	0.849
65	0.077	0.405	0.255	0.184	0.211	0.241	0.275	0.313	0.356	0.46	0.521	0.59	0.668	0.754	0.851
66	0.078	0.408	0.254	0.186	0.213	0.243	0.277	0.316	0.359	0.463	0.524	0.593	0.67	0.757	0.854
67	0.078	0.41	0.252	0.188	0.215	0.245	0.279	0.318	0.361	0.465	0.527	0.596	0.673	0.759	0.856
68	0.079	0.412	0.251	0.19	0.216	0.247	0.281	0.32	0.363	0.467	0.529	0.597	0.675	0.761	0.857
69	0.081	0.414	0.25	0.191	0.218	0.248	0.283	0.321	0.365	0.468	0.53	0.599	0.675	0.761	0.857
70	0.085	0.415	0.249	0.192	0.219	0.25	0.284	0.323	0.366	0.469	0.531	0.599	0.675	0.76	0.855
71	0.091	0.416	0.247	0.193	0.22	0.251	0.285	0.324	0.367	0.47	0.531	0.599	0.674	0.759	0.852
72	0.098	0.416	0.246	0.193	0.221	0.251	0.286	0.324	0.368	0.47	0.531	0.598	0.673	0.756	0.849
73	0.104	0.417	0.245	0.194	0.221	0.252	0.287	0.325	0.368	0.471	0.531	0.597	0.672	0.754	0.845
74	0.108	0.417	0.243	0.195	0.222	0.253	0.287	0.326	0.369	0.471	0.53	0.597	0.67	0.752	0.842
75	0.11	0.418	0.242	0.196	0.223	0.254	0.288	0.327	0.37	0.471	0.53	0.596	0.669	0.75	0.84
76	0.108	0.418	0.241	0.197	0.224	0.255	0.289	0.327	0.37	0.471	0.53	0.596	0.668	0.749	0.838
77	0.104	0.418	0.24	0.198	0.225	0.256	0.29	0.328	0.371	0.471	0.53	0.596	0.668	0.748	0.837
78	0.098	0.419	0.239	0.199	0.227	0.257	0.291	0.329	0.372	0.472	0.53	0.596	0.668	0.748	0.837
79	0.089	0.419	0.238	0.201	0.228	0.258	0.292	0.33	0.372	0.472	0.531	0.596	0.668	0.748	0.837
80	0.079	0.42	0.236	0.202	0.229	0.259	0.293	0.331	0.373	0.472	0.531	0.596	0.668	0.748	0.837
81	0.069	0.42	0.235	0.204	0.231	0.261	0.294	0.332	0.374	0.473	0.531	0.596	0.668	0.748	0.838
82	0.058	0.421	0.234	0.205	0.232	0.262	0.295	0.332	0.374	0.473	0.531	0.596	0.668	0.748	0.838
83	0.049	0.421	0.233	0.207	0.233	0.263	0.296	0.333	0.375	0.473	0.531	0.596	0.668	0.748	0.837
84	0.039	0.422	0.231	0.209	0.235	0.264	0.297	0.334	0.375	0.473	0.531	0.595	0.667	0.747	0.837
85	0.031	0.422	0.23	0.21	0.236	0.265	0.298	0.335	0.376	0.473	0.531	0.595	0.667	0.747	0.836
86	0.022	0.422	0.229	0.212	0.238	0.267	0.299	0.336	0.377	0.474	0.531	0.595	0.666	0.746	0.835

**TABLE 5:** Age‐specific LMS parameters and FM/FFM *z* scores for females.

Age	L	M	S	−3.0 SD	−2.5 SD	−2.0 SD	−1.5 SD	−1.0 SD	−0.5 SD	0.5 SD	1.0 SD	1.5 SD	2.0 SD	2.5 SD	3.0 SD
45	0.209	0.573	0.286	0.222	0.264	0.311	0.365	0.426	0.495	0.66	0.757	0.864	0.984	1.116	1.262
46	0.256	0.579	0.285	0.22	0.263	0.312	0.368	0.43	0.5	0.666	0.762	0.869	0.986	1.114	1.255
47	0.303	0.585	0.284	0.218	0.263	0.313	0.371	0.435	0.506	0.672	0.768	0.873	0.988	1.113	1.248
48	0.347	0.591	0.283	0.216	0.263	0.315	0.374	0.439	0.511	0.679	0.774	0.878	0.991	1.112	1.243
49	0.388	0.598	0.281	0.215	0.263	0.317	0.377	0.444	0.517	0.686	0.781	0.884	0.994	1.113	1.24
50	0.422	0.605	0.279	0.215	0.265	0.32	0.382	0.45	0.524	0.693	0.788	0.89	0.999	1.116	1.24
51	0.451	0.613	0.277	0.216	0.267	0.324	0.387	0.456	0.531	0.701	0.796	0.897	1.005	1.12	1.241
52	0.472	0.621	0.274	0.219	0.271	0.329	0.393	0.463	0.539	0.709	0.804	0.904	1.012	1.125	1.245
53	0.488	0.629	0.272	0.223	0.276	0.335	0.4	0.47	0.547	0.718	0.812	0.912	1.019	1.131	1.249
54	0.5	0.638	0.268	0.228	0.282	0.342	0.407	0.478	0.555	0.726	0.82	0.92	1.025	1.137	1.254
55	0.511	0.646	0.264	0.234	0.289	0.349	0.415	0.486	0.564	0.734	0.828	0.927	1.032	1.142	1.257
56	0.522	0.654	0.26	0.24	0.296	0.357	0.423	0.495	0.572	0.742	0.835	0.933	1.037	1.146	1.259
57	0.535	0.662	0.256	0.246	0.303	0.364	0.431	0.503	0.58	0.75	0.842	0.939	1.041	1.148	1.26
58	0.549	0.67	0.252	0.252	0.309	0.371	0.439	0.511	0.588	0.757	0.848	0.944	1.045	1.15	1.26
59	0.565	0.677	0.248	0.258	0.315	0.378	0.446	0.518	0.595	0.763	0.854	0.949	1.048	1.152	1.26
60	0.582	0.683	0.245	0.262	0.32	0.384	0.452	0.524	0.602	0.769	0.859	0.953	1.051	1.153	1.259
61	0.601	0.688	0.242	0.265	0.325	0.389	0.457	0.53	0.607	0.774	0.863	0.956	1.053	1.153	1.257
62	0.62	0.693	0.239	0.268	0.328	0.393	0.462	0.535	0.612	0.778	0.866	0.959	1.054	1.153	1.255
63	0.641	0.697	0.237	0.269	0.33	0.396	0.465	0.539	0.616	0.781	0.869	0.96	1.055	1.152	1.253
64	0.661	0.7	0.236	0.27	0.332	0.398	0.468	0.542	0.619	0.784	0.871	0.961	1.055	1.151	1.25
65	0.681	0.702	0.234	0.27	0.333	0.4	0.47	0.544	0.622	0.786	0.872	0.962	1.054	1.149	1.246
66	0.7	0.704	0.232	0.271	0.334	0.401	0.472	0.546	0.624	0.787	0.873	0.961	1.053	1.146	1.242
67	0.717	0.705	0.231	0.271	0.335	0.402	0.473	0.548	0.625	0.788	0.873	0.96	1.05	1.143	1.237
68	0.733	0.706	0.23	0.271	0.335	0.403	0.474	0.549	0.626	0.788	0.872	0.959	1.048	1.139	1.232
69	0.748	0.706	0.228	0.27	0.335	0.404	0.475	0.549	0.626	0.787	0.871	0.957	1.045	1.135	1.227
70	0.762	0.705	0.227	0.27	0.335	0.404	0.476	0.55	0.626	0.787	0.87	0.955	1.042	1.131	1.221
71	0.776	0.705	0.226	0.269	0.335	0.404	0.476	0.55	0.626	0.785	0.868	0.952	1.038	1.126	1.215
72	0.79	0.704	0.225	0.268	0.334	0.404	0.475	0.549	0.626	0.784	0.866	0.95	1.035	1.121	1.209
73	0.806	0.703	0.224	0.267	0.333	0.403	0.475	0.549	0.625	0.782	0.864	0.946	1.031	1.116	1.203
74	0.822	0.701	0.224	0.264	0.332	0.402	0.474	0.548	0.624	0.781	0.861	0.943	1.026	1.111	1.197
75	0.839	0.7	0.223	0.262	0.33	0.4	0.472	0.546	0.622	0.778	0.858	0.94	1.022	1.105	1.19
76	0.858	0.698	0.223	0.259	0.327	0.398	0.471	0.545	0.621	0.776	0.855	0.936	1.017	1.1	1.183
77	0.877	0.695	0.222	0.255	0.325	0.396	0.469	0.543	0.619	0.773	0.852	0.932	1.012	1.093	1.175
78	0.898	0.693	0.222	0.252	0.322	0.394	0.467	0.541	0.617	0.77	0.849	0.927	1.007	1.087	1.168
79	0.919	0.691	0.221	0.248	0.319	0.391	0.465	0.539	0.615	0.767	0.845	0.923	1.001	1.08	1.16
80	0.942	0.688	0.221	0.243	0.315	0.389	0.462	0.537	0.612	0.764	0.841	0.918	0.995	1.073	1.151
81	0.967	0.685	0.22	0.239	0.312	0.386	0.46	0.535	0.61	0.761	0.837	0.913	0.989	1.066	1.142
82	0.992	0.682	0.22	0.234	0.308	0.383	0.458	0.533	0.607	0.758	0.833	0.908	0.983	1.058	1.133
83	1.018	0.68	0.219	0.229	0.305	0.38	0.455	0.53	0.605	0.754	0.829	0.903	0.977	1.051	1.125
84	1.045	0.677	0.219	0.224	0.301	0.377	0.453	0.528	0.603	0.751	0.824	0.898	0.971	1.044	1.116
85	1.071	0.674	0.218	0.218	0.297	0.375	0.451	0.526	0.601	0.748	0.821	0.893	0.965	1.036	1.108
86	1.098	0.672	0.218	0.213	0.293	0.372	0.448	0.524	0.598	0.745	0.817	0.888	0.959	1.029	1.099

Graphical assessment of model fit showed evidence of adequate model fit and normality of the generated percentile curves (Figures S1–S4). We additionally compared weighted and unweighted percentile curves as a sensitivity analysis, and results were consistent with primary analyses (Figure S5).

## 4 Discussion

This study presents age‐ and sex‐specific FM/FFM percentile curves using a large population‐based cohort of older adults in Canada. Our percentile curves describe the population‐level distribution of sarcopenic obesity in Canada and differences in sarcopenic obesity according to age and sex. Results demonstrate that men have lower FM/FFM values than females at all selected percentile values. FM/FFM percentile curves for men changed less across the lifespan than for women and plateaued at lower values. On average, individuals gained fat mass and lost muscle mass with age, resulting in a downward trend in FM/FFM percentile curves at older ages. These changes are potentially a result of reductions in resting metabolic rate and macronutrient oxidation that occur with age [24].

Our findings are largely consistent with previous studies reporting age‐ and sex‐specific FM/FFM percentiles for study populations from the United States (US) and China [6, 25, 26]. Based on whole‐body DXA measurements data from 13 236 (6656 males and 6580 females) US adults aged 18 to 85 years from the 1999–2004 National Health and Nutrition Examination Survey (NHANES), Siervo et al. reported a maximum 50th percentile FM/FFM value of 0.46 at age 74 years for males and 0.76 at age 66 years for females, and a maximum 95th percentile value of 0.65 at age 70 years for males and 1.04 at age 60 years for females [26]. Similarly, based on bioelectrical impedance analysis (BIA) data from 15 802 Chinese adults (6334 males and 9468 females) aged 35 to 74 years from the Ningxia cohort study, Zhang et al. reported a maximum 50th percentile FM/FFM value of 0.42 at age 70 years or older for males and 0.64 at age 70 years or older for females, and a maximum 98th percentile value of 0.75 at age 70 years or older for males and 1.02 at age 70 years or older for females [25].

Although Siervo et al. and Zhang et al. created FM/FFM percentile curves using populations in the US and Western China, the present study extends their work in several important ways. Data from a Canadian context offer valuable normative data for populations with distinct demographic and health profiles not previously captured in existing studies [27]. The present findings suggest that the body composition of Canadian older adults is similar to US older adults. Canadian older adults appear to have higher FM/FFM values compared with Chinese older adults. This is an interesting finding because it has been shown that some Asian populations have higher body fat and lower lean mass, potentially due to hormonal or genetic factors [28]. It is important to note that the measurement approaches for body composition differed across these studies, and that could, in part, explain the different findings. Zhang and colleagues used BIA, whereas in the present work, DXA measurements were used to examine body composition [29].

Prior research has demonstrated the prevalence of sarcopenic obesity increases with age and is more prevalent in women [30]. We reported notable sex differences in FM/FFM, with females having higher FM/FFM values than males. This is consistent with sexual dimorphism in body composition [31]. Sexual dimorphism describes the different biological traits between males and females and is known to begin at birth and to continue into adulthood [32]. This results in adult males having greater total lean mass and lower fat mass than adult females, hence lower FM/FFM values. Sex hormones are known to be a major factor contributing to sexual dimorphism. Oestrogen affects adipose tissue accumulation, metabolism and distribution, whereas testosterone leads to increased lean mass [31]. Sex hormones may also explain the rapid changes in FM/FFM percentiles with age for females compared with males. Females experience significant changes in sex hormones during the menopausal transition, leading to changes in body composition, an increase in fat mass and a reduction in fat‐free mass [33]. At the cellular level, sexual dimorphism is thought to be dictated by differences in sex chromosomes leading to sex‐specific gene expression [34]. At the population level, this phenomenon is manifested as higher prevalence of obesity in females than males [35]. Taken together, the present findings highlight the importance of age‐ and sex‐related differences in FM/FFM percentiles among older adults.

From a physiological perspective, FM/FFM directly operationalises the balance between metabolic load and metabolic capacity under the load‐capacity model proposed by Prado and colleagues [11]. Metabolic load, represented by fat mass, is associated with characteristics such as inflammation, lipid accumulation and metabolic demands, whereas metabolic capacity, represented by fat‐free mass, is associated with glucose regulation, lipid metabolism and metabolic function [36]. A higher FM/FFM therefore reflects a state in which metabolic load disproportionately exceeds metabolic capacity, a condition that is central to the pathophysiology of sarcopenic obesity [11, 36]. Compared with conventional measures of sarcopenic obesity that define obesity and sarcopenia using independent cut points, FM/FFM provides an integrated assessment of body composition, taking into account the joint effect of fat mass and fat‐free mass [6, 11]. Individuals with similar absolute fat or fat‐free mass values may differ significantly in metabolic and functional risk depending on their relative joint distribution [11]. FM/FFM addresses this limitation by providing a continuous measure that inherently scales fat mass to the amount of metabolically active tissue, thereby reflecting the relative burden placed on fat‐free mass [11]. An important contribution of the present work expressing FM/FFM as age‐ and sex‐specific percentiles is that it allows for inference about an individual's metabolic state relative to others of the same age and sex in the same population.

The present study has several strengths and limitations. First, to our knowledge, this is the first study to develop age‐ and sex‐specific FM/FFM percentiles for Canadian older adults using DXA. DXA is a gold‐standard measure of body composition that quantifies the attenuation of low‐emission X‐ray beams passing through body tissues and is considered the reference method for assessing body composition [29]. Secondly, this study used a large, nationally representative dataset, which adds to the generalisability of its findings. Although it is common to define older adults as individuals aged 65 years or older, an important strength of the current research was our ability to include participants in midlife (45–64 years). Age‐related change in body composition is known to begin in midlife, and these data allow us to capture changes that are relevant to the development of sarcopenic obesity in later life [24, 37].

The present work also includes several limitations. The majority of CLSA participants identified as white. Future research is needed to investigate racial and ethnic differences in FM/FFM percentiles. Additionally, survivor bias may be a concern; those included in the present study could have been individuals healthy enough to be alive at study enrolment. However, this concern is allayed by the fact that CLSA recruits participants starting in midlife (45 years). Finally, we described sarcopenic obesity using FM/FFM and did not incorporate a measure of muscle function into the definition. Several authors have noted muscle function is an important component of sarcopenic obesity, as evidenced by current definitions of sarcopenic obesity that incorporate functional parameters (e.g., gait speed or hand grip strength) [5]. Future research should consider whether the muscle function could also be integrated into FM/FFM percentiles. Deterioration in muscle function may reflect downstream consequences of metabolic imbalance [38].

In the future, FM/FFM percentiles could be used as a screening tool to identify patients in need of intervention before measurable declines in muscle mass occur. Additional research is needed to examine changes in FM/FFM percentile curves in response to specific interventions for sarcopenic obesity. The FM/FFM percentile curves developed in the present work incorporate information on age, sex, obesity and sarcopenia into one metric. This work has significant potential for researchers interested in investigating sarcopenic obesity‐related health outcomes at the population level.

## Funding

Muscle Mass in the Life and Longevity After Cancer (LILAC) study (5R37CA258761; PI: Hailey R. Banack) funded by the National Institutes Of Health (NIH) and National Cancer Institute (NCI).

## Disclosure

The opinions expressed in this manuscript are the author's own and do not reflect the views of the Canadian Longitudinal Study on Aging.

## Ethics Statement

This work was approved by the University of Toronto Research Ethics Board and in compliance with the 1964 Declaration of Helsinki and its later amendments.

## Conflicts of Interest

The authors declare no conflicts of interest.

## Supporting information


**Table S1:** AIC and BIC values from LMS procedure—male.
**Table S2:** AIC and BIC values from LMS procedure—female.
**Figure S1:** Q‐Q plot of LMS Z‐scores—male.
**Figure S2:** Q‐Q plot of LMS Z‐scores—female.
**Figure S3:** Observed versus predicted LMS percentiles (5th, 50th and 95th)—male.
**Figure S4:** Observed versus predicted LMS percentiles (5th, 50th and 95th)—female.
**Figure S5:** FM/FFM percentiles—unweighted.

## Data Availability

Data are available from the Canadian Longitudinal Study on Aging (www.clsa‐elcv.ca) for researchers who meet the criteria for access to de‐identified CLSA data.
