# Supplementary material for: Age‐ and Sex‐Specific Percentile Curves for the FM/FFM Ratio Using Data From the Canadian Longitudinal Study on Aging
Source: J Cachexia Sarcopenia Muscle. 2026 Oct 8;17(5):e70399. doi: 10.1002/jcsm.70399 (PMC13650270; doi:10.1002/jcsm.70399)
Supplement: Supplementary file 1 [file JCSM-17-e70399-s001.docx]

**SUPPLEMENTARY MATERIAL**

**Table S1. AIC and BIC Values from LMS Procedure – Male**

| **df (M)** | **df (S)** | **df (L)** | **AIC** | **BIC** |
| --- | --- | --- | --- | --- |
| **3** | **3** | **3** | **-21983.36887** | **-21862.44924** |
| **4** | **3** | **3** | **-21985.05287** | **-21856.57596** |
| **5** | **3** | **3** | **-21986.69474** | **-21850.66013** |
| **6** | **3** | **3** | **-21988.22251** | **-21844.63094** |
| **3** | **4** | **3** | **-21982.16761** | **-21853.69089** |
| **4** | **4** | **3** | **-21983.83525** | **-21847.80101** |
| **5** | **4** | **3** | **-21985.46645** | **-21841.87438** |
| **6** | **4** | **3** | **-21986.98963** | **-21835.84084** |
| **3** | **5** | **3** | **-21981.31303** | **-21845.27841** |
| **4** | **5** | **3** | **-21982.96109** | **-21839.36929** |
| **5** | **5** | **3** | **-21984.58012** | **-21833.43067** |
| **6** | **5** | **3** | **-21986.0959** | **-21827.3893** |
| **3** | **6** | **3** | **-21980.70326** | **-21837.11117** |
| **4** | **6** | **3** | **-21982.33387** | **-21831.18449** |
| **5** | **6** | **3** | **-21983.94116** | **-21825.234** |
| **6** | **6** | **3** | **-21985.45062** | **-21819.18666** |
| **3** | **3** | **4** | **-21983.18395** | **-21854.70682** |
| **4** | **3** | **4** | **-21984.74075** | **-21848.70631** |
| **5** | **3** | **4** | **-21986.30552** | **-21842.71345** |
| **6** | **3** | **4** | **-21987.80146** | **-21836.65232** |
| **3** | **4** | **4** | **-21981.97724** | **-21845.94279** |
| **4** | **4** | **4** | **-21983.51887** | **-21839.92716** |
| **5** | **4** | **4** | **-21985.07431** | **-21833.92492** |
| **6** | **4** | **4** | **-21986.56521** | **-21827.85878** |
| **3** | **5** | **4** | **-21981.11561** | **-21837.52352** |
| **4** | **5** | **4** | **-21982.63899** | **-21831.48961** |
| **5** | **5** | **4** | **-21984.18281** | **-21825.47595** |
| **6** | **5** | **4** | **-21985.66819** | **-21819.40424** |
| **3** | **6** | **4** | **-21980.50041** | **-21829.35082** |
| **4** | **6** | **4** | **-21982.00649** | **-21823.29976** |
| **5** | **6** | **4** | **-21983.53948** | **-21817.275** |
| **6** | **6** | **4** | **-21985.01961** | **-21811.19807** |
| **3** | **3** | **5** | **-21983.09599** | **-21847.06126** |
| **4** | **3** | **5** | **-21984.55257** | **-21840.96067** |
| **5** | **3** | **5** | **-21986.05973** | **-21834.91019** |
| **6** | **3** | **5** | **-21987.53598** | **-21828.82925** |
| **3** | **4** | **5** | **-21981.88026** | **-21838.28809** |
| **4** | **4** | **5** | **-21983.32333** | **-21832.17408** |
| **5** | **4** | **5** | **-21984.82248** | **-21826.11553** |
| **6** | **4** | **5** | **-21986.29518** | **-21820.0313** |
| **3** | **5** | **5** | **-21981.00853** | **-21829.85881** |
| **4** | **5** | **5** | **-21982.43444** | **-21823.72747** |
| **5** | **5** | **5** | **-21983.92293** | **-21817.65843** |
| **6** | **5** | **5** | **-21985.39056** | **-21811.56881** |
| **3** | **6** | **5** | **-21980.38557** | **-21821.67827** |
| **4** | **6** | **5** | **-21981.7947** | **-21815.53021** |
| **5** | **6** | **5** | **-21983.27309** | **-21809.45094** |
| **6** | **6** | **5** | **-21984.73625** | **-21803.35724** |
| **3** | **3** | **6** | **-21983.0285** | **-21839.4364** |
| **4** | **3** | **6** | **-21984.42117** | **-21833.27157** |
| **5** | **3** | **6** | **-21985.89331** | **-21827.18613** |
| **6** | **3** | **6** | **-21987.36102** | **-21821.09686** |
| **3** | **4** | **6** | **-21981.80345** | **-21830.65373** |
| **4** | **4** | **6** | **-21983.18384** | **-21824.47719** |
| **5** | **4** | **6** | **-21984.64901** | **-21818.38441** |
| **6** | **4** | **6** | **-21986.1143** | **-21812.29294** |
| **3** | **5** | **6** | **-21980.92217** | **-21822.21512** |
| **4** | **5** | **6** | **-21982.28624** | **-21816.02176** |
| **5** | **5** | **6** | **-21983.74168** | **-21809.91972** |
| **6** | **5** | **6** | **-21985.20264** | **-21803.82363** |
| **3** | **6** | **6** | **-21980.29316** | **-21814.02865** |
| **4** | **6** | **6** | **-21981.64079** | **-21807.819** |
| **5** | **6** | **6** | **-21983.08618** | **-21801.70675** |
| **6** | **6** | **6** | **-21984.54365** | **-21795.60716** |

**Table S2. AIC and BIC Values from LMS Procedure - Female**

| **df (M)** | **df (S)** | **df (L)** | **AIC** | **BIC** |
| --- | --- | --- | --- | --- |
| **3** | **3** | **3** | **-11855.62296** | **-11734.15782** |
| **4** | **3** | **3** | **-11858.5863** | **-11729.5295** |
| **5** | **3** | **3** | **-11860.22353** | **-11723.57524** |
| **6** | **3** | **3** | **-11861.08443** | **-11716.84441** |
| **3** | **4** | **3** | **-11856.88236** | **-11727.82548** |
| **4** | **4** | **3** | **-11860.04413** | **-11723.3955** |
| **5** | **4** | **3** | **-11861.78979** | **-11717.54974** |
| **6** | **4** | **3** | **-11862.68617** | **-11710.85461** |
| **3** | **5** | **3** | **-11857.53858** | **-11720.88997** |
| **4** | **5** | **3** | **-11860.80602** | **-11716.56613** |
| **5** | **5** | **3** | **-11862.58314** | **-11710.7515** |
| **6** | **5** | **3** | **-11863.4714** | **-11704.04837** |
| **3** | **6** | **3** | **-11857.87782** | **-11713.6378** |
| **4** | **6** | **3** | **-11861.18865** | **-11709.35716** |
| **5** | **6** | **3** | **-11862.96101** | **-11703.53791** |
| **6** | **6** | **3** | **-11863.818** | **-11696.80322** |
| **3** | **3** | **4** | **-11854.87083** | **-11725.81407** |
| **4** | **3** | **4** | **-11857.57339** | **-11720.925** |
| **5** | **3** | **4** | **-11859.04245** | **-11714.80244** |
| **6** | **3** | **4** | **-11859.80846** | **-11707.97689** |
| **3** | **4** | **4** | **-11856.08609** | **-11719.43761** |
| **4** | **4** | **4** | **-11858.98905** | **-11714.74901** |
| **5** | **4** | **4** | **-11860.56028** | **-11708.72856** |
| **6** | **4** | **4** | **-11861.36279** | **-11701.93959** |
| **3** | **5** | **4** | **-11856.71264** | **-11712.47261** |
| **4** | **5** | **4** | **-11859.71815** | **-11707.88651** |
| **5** | **5** | **4** | **-11861.32697** | **-11701.90378** |
| **6** | **5** | **4** | **-11862.11847** | **-11695.1037** |
| **3** | **6** | **4** | **-11857.0459** | **-11705.2143** |
| **4** | **6** | **4** | **-11860.08948** | **-11700.66629** |
| **5** | **6** | **4** | **-11861.69275** | **-11694.67796** |
| **6** | **6** | **4** | **-11862.45486** | **-11687.8485** |
| **3** | **3** | **5** | **-11853.76057** | **-11717.11196** |
| **4** | **3** | **5** | **-11856.31098** | **-11712.07093** |
| **5** | **3** | **5** | **-11857.67285** | **-11705.84125** |
| **6** | **3** | **5** | **-11858.38345** | **-11698.96053** |
| **3** | **4** | **5** | **-11854.9499** | **-11710.70988** |
| **4** | **4** | **5** | **-11857.6984** | **-11705.86665** |
| **5** | **4** | **5** | **-11859.16748** | **-11699.74417** |
| **6** | **4** | **5** | **-11859.91081** | **-11692.89603** |
| **3** | **5** | **5** | **-11855.56562** | **-11703.73401** |
| **4** | **5** | **5** | **-11858.41375** | **-11698.99041** |
| **5** | **5** | **5** | **-11859.91956** | **-11692.90455** |
| **6** | **5** | **5** | **-11860.65617** | **-11686.04972** |
| **3** | **6** | **5** | **-11855.90067** | **-11696.47747** |
| **4** | **6** | **5** | **-11858.7874** | **-11691.77256** |
| **5** | **6** | **5** | **-11860.28919** | **-11685.6827** |
| **6** | **6** | **5** | **-11860.99694** | **-11678.79891** |
| **3** | **3** | **6** | **-11852.41463** | **-11708.17464** |
| **4** | **3** | **6** | **-11854.88651** | **-11703.05494** |
| **5** | **3** | **6** | **-11856.19665** | **-11696.77348** |
| **6** | **3** | **6** | **-11856.87741** | **-11689.86268** |
| **3** | **4** | **6** | **-11853.59241** | **-11701.7608** |
| **4** | **4** | **6** | **-11856.26388** | **-11696.84049** |
| **5** | **4** | **6** | **-11857.68021** | **-11690.66541** |
| **6** | **4** | **6** | **-11858.39645** | **-11683.79008** |
| **3** | **5** | **6** | **-11854.21207** | **-11694.78887** |
| **4** | **5** | **6** | **-11856.9801** | **-11689.96532** |
| **5** | **5** | **6** | **-11858.43375** | **-11683.82737** |
| **6** | **5** | **6** | **-11859.14294** | **-11676.94499** |
| **3** | **6** | **6** | **-11854.55421** | **-11687.53936** |
| **4** | **6** | **6** | **-11857.36313** | **-11682.75677** |
| **5** | **6** | **6** | **-11858.81467** | **-11676.6167** |
| **6** | **6** | **6** | **-11859.49704** | **-11669.70752** |

**Figure S1. Q-Q Plot of LMS Z-scores - Male**

**Figure S2. Q-Q Plot of LMS Z-scores - Female**

**Figure S3. Observed vs Predicted LMS Percentiles (5^th^, 50^th^, and 95^th^) - Male**

**Figure S4. Observed vs Predicted LMS Percentiles (5^th^, 50^th^, and 95^th^) - Female**

**Figure S5. FM/FFM Percentiles – Unweighted**
